# Editorial: Case reports in psychopharmacology, volume II

**DOI:** 10.3389/fpsyt.2025.1554037

**Published:** 2025-02-13

**Authors:** Matej Stuhec, Patricia Di Ciano

**Affiliations:** ^1^ Faculty of Medicine, University of Maribor, Maribor, Slovenia; ^2^ Department of Clinical Pharmacy, Ormoz Psychiatric Hospital, Ormoz, Slovenia; ^3^ Institute for Mental Health Policy Research, Centre for Addiction and Mental Health, Toronto, ON, Canada; ^4^ Department of Pharmacology and Toxicology, University of Toronto, Toronto, ON, Canada; ^5^ Dalla Lana School of Public Health, University of Toronto, Toronto, ON, Canada; ^6^ Campbell Family Mental Health Research Institute, Toronto, ON, Canada

**Keywords:** major depressive disorder, psychosis, bipolar disorder, adverse events, post-traumatic stress disorder

This is the second volume of *Case Reports in Psychopharmacology*. This volume includes a diverse set of papers reporting on the treatment of Psychiatric disorders, including post-traumatic stress disorder, bipolar disorder, major depressive disorder and schizophrenia. The approaches range from pharmacotherapies to pharmacogenetics and the findings present new considerations for treatments. Unlike our previous volume, the reports contained herein all focus on adverse events and present considerations for the safe use of these therapies.

In a report by Wei at al., the authors report on hypothyroidism and sinus dysfunction following use of lithium and paliperidone to treat bipolar disorder with psychotic symptoms. Lithium is commonly used to treat bipolar disorder, and it is often associated with hypothyroidism. Paliperidone, which is not associated with hypothyroidism, is not regularly used in patients under 18 years of age. In this report, a single 17-year old patient was treated off-label with a combination of lithium (500 mg twice daily) and paliperidone (9 mg once daily; titrated down to 6 mg after 2 months). The patient had normal thyroid and heart rate function prior to treatment. After treatment she presented with thyroid abnormalities that were considered subclinical. The patient was bradycardic at 41 beats per minute and blood pressure was 95/57 mmHg; an electrocardiogram revealed bradycardia. The patient’s thyroid function normalized 20 days after stopping these medications. Bradycardia and sinus dysfunction also normalized within this timeframe. This is the first report of subclinical hypothyroidism and bradycardis with QT prolongation associated with lithium and paliperidone treatment.

In a case report by Hudnik at al., a pharmacogenetic approach is described for the treatment of psychotic symptoms. Normally, treatment for schizophrenia and mood disorders is based on a ‘treatment as usual’ approach with antipsychotics in which the same types of treatment are given to patients. This can lead to lengthy dose adjustments and unwanted adverse drug reactions. Pharmacogenetic approaches can be applied to tailor medications based on an individual patient’s genes involved in the metabolism of antipsychotics in the liver. In this case report, a 60 year old patient with psychotic symptoms presented with severe extrapyramidal symptoms and malignant neuroleptic syndrome after treatment with risperidone and a variety of other antipsychotics. Pharmacogenetic analysis of gene variants of CYP2D6, CYP3A4, CYP1A2, CYP3A5, ABCB1 and ABCG2 revealed a specific metabolic profile, and when treatment was tailored to the patient’s individual profile, the adverse reactions were less severe and the psychotic symptoms disappeared.

3,4-methylenedioxymethamphetamine (MDMA) has been increasingly studied as an adjunct for the treatment of post-traumatic stress disorder. Given the extensive hepatic excretion of MDMA, concerns have been raised over the potential of MDMA to lead to hepatic damage. However, given the rare occurrence of this adverse reaction, it is difficult to capture in clinical trials. In a study by Makunts and Abagyan, liver injury after treatment was surveyed on the FDA Adverse Event Reporting System, a repository which hosts adverse events submitted to the FDA through MedWatch AE forms 3,500 and 3500A. Of the 1,575 reports selected for analysis from the database, there was a total of 7 with liver failure narrow-FMQ cases. No cases reported MDMA as a ‘primary suspect’ of the adverse events. There were 16 unique liver injury narrow-FMQ adverse events, with one being reported with MDMA as the ‘primary suspect’. The absence of liver injury or liver failure adverse events following treatment with MDMA is consistent with reports from clinical trials.

A study by Zeiss et al. report on the use of esketamine for treatment-resistant depression in two patients. Both patients were emergency cases and esketamine was given as inpatient treatment at 84 mg twice a week in addition to other treatments. For one patient, three days after the fourth dose the patient reported fatigue and muscle pain. A chemical laboratory test confirmed rhabdomyolysis with significantly elevated creatinine kinase (CK), CK-myoglobin and myoglobin levels. The second patient reported muscle weakness after the 10^th^ administration of esketamine. Laboratory tests revealed elevated CK, myoglobin and transaminases. Esketamine was discontinued and laboratory values returned to normal. Depressive symptoms also abated under continued psychotherapy.

Major depressive disorder affects millions, and the first-line treatment includes the selective serotonin reuptake inhibitors. Sertraline is widely prescribed and is generally considered well-tolerated, although some cases of serious adverse events, such as liver toxicity, have been reported. In this case study, Renemane and Rancans report on a 36 year old patient who had been receiving sertraline for 40 days. In the four days prior to admission to hospital the patient experienced nausea, scleral jaundice, vomiting, dark urine, pale stool, epigastric pain, and a subfebrile temperature. Liver function tests revealed acute liver injury. Based on a battery of tests, sertraline-induced acute liver injury was considered as a diagnosis. Sertraline was discontinued abruptly. Liver function parameters approached normal levels by 90 days after discontinuation of sertraline.

Somatic comorbidities are very frequently presented in patients with mental disorders. A collaboration with a clinical pharmacist could be helpful in treatment optimisation. Stuhec et al. studied this aspect in a retrospective pre-post study. The researchers found a positive impact of clinical pharmacists’ interventions on drug-related problems (DRPs) during daily rounds in psychiatric hospitals. The study included 186 patients with mental disorders (mean age: 58.1 years, SD=17.0). During ward rounds, 280 recommendations related to DRPs were conducted (1.5 recommendations per patient). The authors found a high acceptance rate (88.9%), representing a good collaboration between clinical pharmacists and psychiatrists in this hospital. Treatment guidelines adherence improved significantly, meaning clinical pharmacists could be valuable members of the interdisciplinary ward rounds. Although this is a positive study, this practice has not been a standard of care in many countries worldwide (1, 2).
